# Explosive Multiterritorial Arterial Thrombosis Following Brief Heparin Exposure: A Diagnostic Pitfall in the Cath Lab

**DOI:** 10.1155/cric/6999917

**Published:** 2026-08-19

**Authors:** Justine Massart, Claudiu Ungureanu, Yves Dascotte, Jacques Auslender, Adrien Jossart

**Affiliations:** ^1^ Faculty of Medicine and Dentistry, Université Catholique de Louvain (UCLouvain), Brussels, Belgium, uclouvain.be; ^2^ Department of Cardiology, Hôpital de Jolimont, La Louvière, Belgium, jolimont.be

**Keywords:** arterial thrombosis, case report, coronary angiography, heparin-induced thrombocytopenia, HIT type II, ischemia, myocardial infarction, PF4 antibodies

## Abstract

**Background:**

Type II heparin‐induced thrombocytopenia (HIT) is a catastrophic immunothrombotic disorder that can be triggered by minimal heparin exposure during routine coronary angiography.

**Case Summary:**

A 67‐year‐old woman developed “explosive” multiterritorial arterial thrombosis—including acute limb ischemia, an inferior STEMI, and a stroke—within minutes of receiving 5000 units of intra‐arterial heparin. This followed a brief nadroparin exposure 8 days prior. Crucially, events occurred while platelet counts were still normal. Despite a negative functional assay, a high 4T score (6 points) confirmed HIT. Management involved non‐heparin anticoagulation (danaparoid sodium) and thrombectomies.

**Discussion:**

This case highlights a major diagnostic pitfall: severe arterial thrombosis preceding overt thrombocytopenia. The immediate “anaphylactoid‐like” reaction following a heparin bolus is often misidentified as contrast allergy, delaying life‐saving intervention.

**Take Home Messages:**

Suspect HIT in acute systemic reactions or multifocal thrombosis after heparin exposure, regardless of initial platelet counts.

## 1. Introduction

Thrombotic complications occurring during or immediately after diagnostic coronary angiography are rare events [1, 2]. When they do occur, they are mostly related to local procedural factors, including thrombus formation at the level of the arterial sheath, inadequate anticoagulation, or ineffective heparin delivery due to administration through a nonfunctional venous cannula [1].

Standardized anticoagulation protocols using unfractionated heparin are widely implemented during coronary angiography and have markedly reduced the incidence of access‐site thrombosis and catheter‐related thromboembolic events [1]. Nevertheless, even when these protocols are strictly respected, rare and paradoxical clinical scenarios can occur. One such condition is Type II heparin‐induced thrombocytopenia (HIT), a life‐threatening immune‐mediated complication. Unlike benign forms of the disorder, the Type II HIT triggers massive platelet activation, leading to a profound prothrombotic state and a high risk of both venous and arterial thrombosis despite active anticoagulant exposure [2–4].

The reported prevalence of HIT ranges from approximately 0.1% to 5% among patients exposed to heparin, depending on the clinical setting, the type of heparin used, and the duration of exposure [3, 5].

Two distinct forms of HIT are recognized: a benign, nonimmune‐mediated Type I form and a potentially life‐threatening immune‐mediated Type II form associated with thrombotic complications [3, 4]. Once a thrombotic event occurs in the setting of heparin exposure, accurate and timely identification of the underlying mechanism is crucial, as delayed diagnosis of Type II HIT may lead to rapidly progressive, multiterritorial thrombosis with severe clinical consequences [5–8].

We report a case of Type II HIT triggered during a routine diagnostic coronary angiogram, characterized by the acute and simultaneous involvement of multiple vascular territories. Notably, thrombosis occurred before the development of overt thrombocytopenia, and a major diagnostic challenge arose from a discrepancy between a positive screening immunoassay and a negative confirmatory functional assay.

## 2. Case Description

A 67‐year‐old woman with multiple cardiovascular risk factors was admitted for elective coronary angiography. Eight days prior, she had a brief exposure to subcutaneous nadroparin for a suspected pulmonary embolism, which was ruled out. Admission laboratory tests showed no thrombocytopenia.

The right transradial procedure was uneventful, involving 5000 units of intra‐arterial unfractionated heparin. Five minutes post‐examination, the patient developed severe right arm pain, chest tightness, and dyspnea. Initial treatment for a suspected contrast allergy (methylprednisolone and antihistamine) provided no relief.

**Figure 1:**
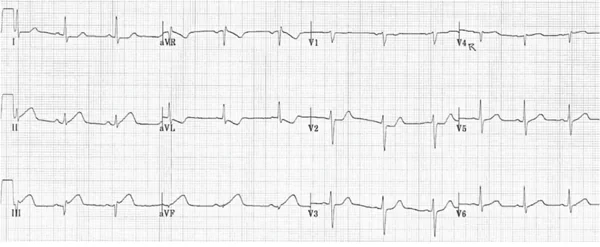
Twelve‐lead electrocardiogram recorded at the onset of acute chest pain, demonstrating an acute inferior ST‐segment elevation myocardial infarction (STEMI), characterized by ST‐segment elevation in Leads II, III, and aVF with reciprocal ST‐segment depression in Leads I and aVL. Prominent ST‐segment depression in the precordial leads (V1–V3) suggests posterior wall extension.

As right limb ischemia progressed, radial artery thrombosis was suspected. However, emergency ultrasound revealed extensive thrombosis of the humeral and ulnar arteries, suggesting a systemic process. During a transfer for an urgent Fogarty thrombectomy, the patient developed an inferior STEMI (Figure 1). An emergent brachial thrombectomy restored ulnar flow; notably, the retrieved thrombus appeared prematurely organized, indicating rapid systemic platelet activation.

**Figure 2:**
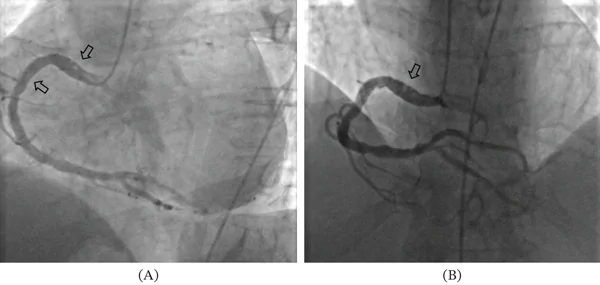
Emergency coronary angiography of the right coronary artery performed via femoral access. (A) Left anterior oblique (LAO) 30° projection clearly showing multiple nonocclusive thrombi (arrows) within the proximal and middle segments of the vessel, highlighted as prominent intraluminal filling defects. (B) Cranial 30° projection demonstrating intraluminal filling defects in the proximal and middle segments.

Because of persistent ST‐elevation and a high 4T score of 6 (Table 1), repeat angiography was performed via femoral access, strictly avoiding heparin. This confirmed nonocclusive thrombi in the right coronary artery (Figure 2, Video 1, Video 2). Anticoagulation was switched to danaparoid sodium.

**Table 1:** The 4T pretest clinical probability score for HIT [9].

Criteria	2 points	1 point	0 point
Thrombocytopenia	Platelet count fall > 50% AND platelet nadir ≥ 20 × 10^9^/L	Platelet count fall 30%–50% OR platelet nadir 10–19 x 10^9^/L	Platelet count fall < 30% OR platelet nadir < 10 × 10^9^/L
Timing of platelet count fall	Clear onset between Days 5 and 10 OR ≤ 1 day (if heparin exposure within past 30 days)	Consistent with days 5–10 fall, but not clear; onset after Day 10; OR ≤ 1 day (if heparin exposure 31–100 days ago)	Platelet count fall < 4 days without recent exposure
Thrombosis or other sequelae	Confirmed new thrombosis; skin necrosis; OR acute systemic reaction after IV heparin bolus	Progressive or recurrent thrombosis; non‐necrotizing skin lesions; OR suspected thrombosis	None
Other causes for thrombocytopenia	None evident	Possible	Definite

*Note:* Interpretation of Total Score: 6–8 points: High probability of HIT (approx. 64%); 4–5 points: Intermediate probability (approx. 14%); 0–3 points: Low probability (approx. < 1%).

The clinical course included (Table 2) the following:

• Hematological Profile: Platelets dropped 65% (from 271,000 to 95,000/mm^3^). Anti‐PF4 antibodies were positive, though the functional assay was negative. The diagnosis of Type II HIT was maintained based on clinical probability.
• Complications: The patient suffered a right occipital ischemic stroke and recurrent right upper limb ischemia despite danaparoid therapy. A repeat surgical thrombectomy via a multi‐access approach was required.
• Management: Anticoagulation was transitioned to rivaroxaban (20 mg/day) before discharge.

**Table 2:** Clinical timeline and platelet count evolution.

Time point	Event	Platelet count (mm^3^)
Day ‐8	Initial exposure: Nadroparin (SC) for suspected PE (ruled out).	Normal (no thrombocytopenia)
Day 0 (AM)	Admission for elective coronary angiography.	271,000 (Baseline)
Day 0 (Procedure)	Intra‐arterial administration of 5000 units of UFH.	—
Day 0 (+5 min)	Acute Event: Severe right arm pain, chest tightness, dyspnea. Initial suspicion of contrast allergy.	—
Day 0 (+1 hour)	Multisystem thrombosis: Acute right arm ischemia and inferior STEMI. Emergent thrombectomy performed.	—
Day 0 (Evening)	Platelet nadir: Confirmation of rapid drop. High 4T score [6] calculated.	95,000 (65% drop).
Day 1	Diagnosis: Positive anti‐PF4 antibodies but negative functional assay; switch to danaparoid.	95,000
Hospital Course	Recurrent limb ischemia, multi‐access thrombectomy, and ischemic stroke.	Gradual recovery
Discharge	Transition to rivaroxaban; persistent limb disability.	341,000

The patient was discharged with persistent functional impairment of the right limb, requiring long‐term physiotherapy.

## 3. Discussion

This case illustrates an uncommon but particularly severe presentation of Type II HIT, triggered by heparin administration during a routine diagnostic coronary angiography (even brief and seemingly low‐risk heparin administration). This case is of significant clinical interest for several reasons:

• Diagnostic Pitfalls: Anaphylactoid Reactions and the Normal Platelet Trap: The clinical presentation posed a significant diagnostic challenge, as the immediate post‐procedure onset of dyspnea, chest tightness, and hypotension was initially misidentified as either a local access complication (spasm, dissection, thrombosis) or a hypersensitivity reaction to the contrast medium. However, the failure of corticosteroids and antihistamines, combined with rapidly progressive acute limb ischemia, pointed toward an aggressive systemic process. This highlights a major clinical pitfall: the early systemic symptoms of Type II HIT can closely mimic anaphylaxis when triggered by a heparin bolus. Crucially, while the classic 4T scoring system specifies an intravenous trigger for these acute systemic reactions (allocating 2 points, Table 1), intra‐arterial administration during coronary angiography is physiologically equivalent. In a sensitized patient, both routes lead to immediate immune complex formation and massive platelet activation. Recognizing this “anaphylactoid‐like” reaction as a manifestation of HIT rather than a contrast allergy is pivotal to avoiding catastrophic treatment delays. At the exact onset of the thrombotic events, the patient’s platelet count was still within the normal range. This case emphasizes that HIT should be suspected based on a relative drop (a 65% decrease from a baseline of 271,000–95,000/mm^3^) rather than waiting for an absolute, severe thrombocytopenic value. Because thrombosis preceded the laboratory’s confirmation of thrombocytopenia, clinical assessment using the 4T score was the only tool sensitive enough to trigger early, life‐saving management.
• Biological Assessment (In Addition to Thrombocytopenia): The diagnostic work‐up revealed positive anti‐PF4 antibodies (screening test) but a negative functional assay (confirmatory test).
  o. Immunoassays, which exist in various formats, measure antibodies against PF4–heparin complexes and serve as the first‐line laboratory test for HIT. While a standard enzyme‐linked immunosorbent assay (ELISA) was utilized to detect anti‐PF4/heparin antibodies, alternative screening modalities include particle gel, lateral flow, or latex assays. They generally have high sensitivity (97%) but have a variably low specificity (30%–80%) [9]. Because of its high negative predictive value, a negative immunoassay effectively excludes HIT. Conversely, a positive result does not confirm the diagnosis owing to its low specificity, thereby necessitating confirmatory testing, typically via a functional assay. Notably, recent data on rapid chemiluminescence enzyme assays (CLIA) show promising results in bridging the gap between functional and immunological assays, offering high diagnostic accuracy with shorter turnaround times [10].
  o. Several types of functional assays are available to directly measure platelet activation induced by anti‐PF4/heparin antibody binding and *F* *c* *γ*RIIa receptor crosslinking. The serotonin release assay (SRA) is the most common functional assay in the diagnosis of HIT. This functional test measures the release of radioactive serotonin from donor platelets upon activation by the patient’s antibodies in the presence of heparin [11]. Other traditional techniques include the heparin‐induced platelet activation (HIPA) test, which relies on a visual assessment of platelet aggregation. In this clinical case, the specific functional modality employed was the heparin‐induced multi‐electrode aggregometry (HIMEA) method [12]. False‐negative functional results are well‐documented across these standard assays; they can arise from technical variations between laboratories, low antibody avidity, or suboptimal timing of sample collection. Therefore, a negative functional assay does not definitively exclude HIT when the clinical probability is high. This observation underscores the need for an integrated clinical–biological approach rather than reliance on a single test. To optimize this detection and overcome the limitations of radioactive or visual aggregation methods, alternative functional modalities have emerged; notably, the determination of HIT can now also be achieved via flow cytometry‐based functional assays, which objectively measure platelet activation by monitoring specific markers such as P‐selectin expression or microparticle formation [13].
• Therapeutic Management: The standard management of HIT involves immediate cessation of all heparin and the initiation of alternative anticoagulants, such as danaparoid, argatroban, or direct oral anticoagulants in the post‐acute phase [2, 8, 9, 14]. A key management point in this case was performing the repeat coronary angiography via femoral access while strictly avoiding heparin based solely on high clinical suspicion. If percutaneous coronary intervention (PCI) is required, direct thrombin inhibitors like bivalirudin or argatroban are superior to danaparoid. Unlike danaparoid, which requires delayed anti‐Xa monitoring and has a long half‐life, these agents allow for real‐time bedside monitoring via activated clotting time (ACT). Bivalirudin is particularly advantageous due to its very short half‐life, ensuring rapid reversibility if further surgery is needed [3]. Finally, systemic thrombolysis was contraindicated here due to the recent surgical thrombectomy, and any unavoidable stenting should prioritize procedural simplicity to minimize heparin‐free time before stable alternative anticoagulation is achieved.

Awareness of these features is critical for interventional cardiologists and other clinicians performing invasive procedures requiring periprocedural anticoagulation, as delayed recognition of HIT may result in catastrophic outcomes.

## 4. Conclusion

Type II HIT is a rare but life‐threatening “immunothrombotic storm” that can be triggered even by minimal heparin exposure during routine angiography. This case highlights that arterial thrombosis often precedes thrombocytopenia, creating a dangerous diagnostic pitfall. Clinicians must look beyond “local” procedural complications—like radial artery thrombosis—when faced with multifocal or systemic events. Because functional assays can be false‐negative, a high 4T score must mandate the immediate cessation of heparin and a switch to alternative anticoagulants to prevent catastrophic outcomes.

## Author Contributions

The clinical management, data retrieval, and case synthesis were initiated by the cardiology team at Hôpital de Jolimont (Dr. Adrien Jossart, Dr. Claudiu Ungureanu, Dr. Yves Dascotte, and Dr. Jacques Auslender). Dr. Justine Massart contributed to the literature review, data extraction, manuscript drafting, and synthesis under the direct supervision of Dr. Adrien Jossart.

## Funding

No funding was received for this manuscript.

## Disclosure

The authors thoroughly reviewed and edited all generated content to ensure scientific accuracy and take full responsibility for the final content of the publication.

## Conflicts of Interest

The authors declare no conflicts of interest.

## Supporting Information

Additional supporting information can be found online in the Supporting Information section.

## Supporting information


**Supporting Information 1** Video 1. Emergency coronary angiography of the right coronary artery performed via right femoral access (30° left anterior oblique projection). The image demonstrates multiple nonocclusive thrombi within the proximal and middle segments, which developed immediately following initial heparin exposure during the diagnostic procedure.

**Supporting Information 2** Video 2. Right coronary angiography via femoral access in a 30° cranial projection.

## Data Availability

The clinical and diagnostic data supporting the findings of this case report are included within the article, its figures, tables, and supporting video files. Additional anonymized clinical details are available from the corresponding author upon reasonable academic request, subject to patient privacy constraints.
